# Longitudinal proliferation mapping *in vivo* reveals NADPH oxidase-mediated dampening of *Staphylococcus aureus* growth rates within neutrophils

**DOI:** 10.1038/s41598-019-42129-6

**Published:** 2019-04-05

**Authors:** Elena A. Seiß, Anna Krone, Pauline Formaglio, Oliver Goldmann, Susanne Engelmann, Burkhart Schraven, Eva Medina, Andreas J. Müller

**Affiliations:** 1 Institute of Molecular and Clinical Immunology, Health Campus Immunology Infectiology and Inflammation (GC-I3), Otto-von-Guericke-University, Leipziger Strasse 44, 39120 Magdeburg, Germany; 2 Infection Immunology, Helmholtz Centre for Infection Research, 38124 Braunschweig, Germany; 3 Microbial Proteomics, Helmholtz Centre for Infection Research, 38124 Braunschweig, Germany; 4 Institute for Microbiology, Technical University Braunschweig, 38106 Braunschweig, Germany; 5 Immune Control, Helmholtz Centre for Infection Research, 38124 Braunschweig, Germany; 6 Intravital Microscopy of Infection and Immunity, Helmholtz Centre for Infection Research, 38124 Braunschweig, Germany

## Abstract

Upon the onset of inflammatory responses, bacterial pathogens are confronted with altered tissue microenvironments which can critically impact on their metabolic activity and growth. Changes in these parameters have however remained difficult to analyze over time, which would be critical to dissect the interplay between the host immune response and pathogen physiology. Here, we established an *in vivo* biosensor for measuring the growth rates of *Staphylococcus aureus* (*S*. *aureus*) on a single cell-level over days in an ongoing cutaneous infection. Using intravital 2-photon imaging and quantitative fluorescence microscopy, we show that upon neutrophil recruitment to the infection site and bacterial uptake, non-lethal dampening of *S*. *aureus* proliferation occurred. This inhibition was supported by NADPH oxidase activity. Therefore, reactive oxygen production contributes to pathogen containment within neutrophils not only by killing *S*. *aureus*, but also by restricting the growth rate of the bacterium.

## Introduction

Distinct bacterial growth rates are decisive for the outcome of infections: First, they impact on susceptibility both to immune effector mechanisms and antibiotic treatment^1,2^. Second, fast-growing bacteria are a source of more and different pathogen-associated molecules than low-proliferating or inactive microbes, thus differentially shaping the immune response^3,4^. Surface proteins and secreted toxins of the Gram-positive *Staphylococcus aureus* (*S*. *aureus*) trigger the production of chemotactic molecules, which results in a massive influx and accumulation of neutrophils at the site of infection^5^. Neutrophils are among the first immune cells in the combat against bacterial infections and contain an impressive set of antimicrobial mechanisms to control Gram-positive bacteria: After phagocytosis, they can kill microbes by antimicrobial peptides in the phagosomes^6^, together with reactive oxygen species (ROS)^7^, which are produced by NADPH oxidase. ROS are also important for catching and killing bacteria via neutrophil extracellular traps (NETs), which comprise chromatin and antimicrobial proteins^8^. Individuals with defects in one of these phagocyte-related defenses suffer from severe and life-threatening bacterial infections^9^. Although *S*. *aureus* has been shown to be able to withstand and counteract innate cellular immune responses^10–12^, it is unclear how neutrophil effector functions impact on the proliferation of the bacterium. Determination of growth rates during infection is of special interest for *S*. *aureus* because slow-growing subpopulations of the pathogen have been proposed to acquire phenotypic resistance against antibiotic treatment, and to give rise to chronic infections due to lower sensitivity to immune-mediated control mechanisms^1,13^. It has however remained difficult to measure bacterial growth rates in the ongoing infection. While a variety of dye-based proliferation measurement approaches have been established for bacteria, the necessity of fluorophore loading or chemical pulsing of fluorescence expression limited the use of most of these systems to only few hours after infection^14–16^.

Here, we established an *in vivo* biosensor for measuring *S*. *aureus* proliferation on a single cell-level at any given time point post infection. This has enabled us to map the bacterial growth rate side by side with the induction of the innate immune response. We show that upon neutrophil recruitment to the site of infection, the bacteria were efficiently taken up into the phagocytes. Concomitantly, *S*. *aureus* growth was significantly reduced, which was aided by functional NADPH oxidase. Taken together, our data suggest that ROS production can contribute to the pathogen containment by non-lethal restriction of *S*. *aureus* proliferation within neutrophils.

## Results

### A biosensor for real-time measurement of *S*. *aureus* growth rate

**Figure 1:**
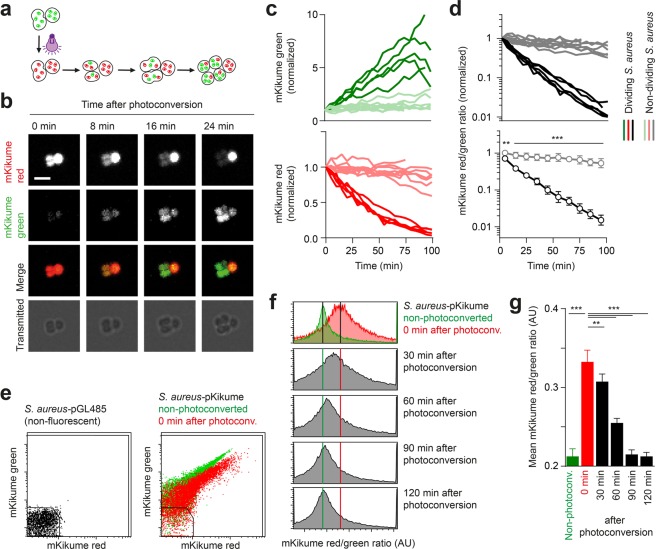
An *in vivo* proliferation reporter system for *S*. *aureus*. (**a**) Schematic representation of the fluorescence-based reporter system. Proliferating bacteria turns green by dilution of the red fluorescent (photoconverted) protein and *de novo* production of green fluorescent mKikume and non-dividing bacteria stays red. (**b**) Confocal time lapse imaging of a photoconverted *S*. *aureus*-pKikume culture growing on an agar pad. Scale bar, 3 µm. (**c**) Normalized red and green fluorescence of dividing (red, green) and non-dividing (pale red and green) bacteria as observed by time lapse imaging on an agar pad. Each curve represents one bacterium followed over up to 100 min. (**d**) Ratio of red to green fluorescence of the measurement shown in (**b**). Black curves, dividing bacteria, grey curves, non-dividing bacteria. Upper panel: Each curve represents one bacterium followed over up to 100 min. Lower panel: Mean +/− standard deviation. ^***^p < 0.001; ^**^p < 0.01 as determined by two-way ANOVA. (**e**) Flow cytometry analysis of non-fluorescent *S*. *aureus* with the empty-vector pGL485 (left) and *S*. *aureus*-pKikume (right) non-photovconverted (green) and 0 min after photoconversion (red). A gate for selecting fluorescent bacteria is indicated. (**f**) Examples of the red to green ratio of fluorescent bacteria in a day culture after photoconversion gated as indicated in (**e**). Green vertical line: distribution maximum of non-photoconverted bacteria. Red vertical line: distribution maximum of photoconverted bacteria. Data in (**e**,**f**) are representative of three individual bacterial cultures. (**g**) Mean (+/− standard deviation) mKikume red to green fluorescence ratio development over time of three individual day cultures after photoconversion. ^***^p < 0.001; ^**^p < 0.01 as determined by one-way ANOVA.

It has been difficult so far to determine *S*. *aureus* growth rates *in vivo*, especially beyond the first few rounds of cell division after inoculation^15,16^. Therefore, we generated a proliferation biosensor based on dilution and *de novo* production of a photoconvertible fluorescence protein^17,18^. In this approach, the green mKikumeGR^19^ is expressed constitutively from a plasmid (termed pKikume hereafter) with optimized regulatory sequences (see Supplementary methods and Supplementary Fig. S1a–d). The protein could be photoconverted by violet light (Fig. 1a,b and Supplementary Fig. S1e) at any given time point. Thus, we reasoned that bacterial proliferation should be measurable by the recovery from photoconversion through dilution of red (photoconverted) and *de novo* production of green (non-photoconverted) protein taking place in growing, but not in proliferation-inactive bacteria (Fig. 1a). To evaluate the approach, *S*. *aureus* carrying the pKikume reporter plasmid (*S*. *aureus*-pKikume) was examined by time lapse microscopy after photoconversion *in vitro*. Recovery of the original, green fluorescence was detectable after photoconversion in dividing bacteria in a time-dependent manner, whereas the non-dividing bacteria retained the red, photoconverted fluorescence (Fig. 1b,c). Quantitative analysis at a single bacteria level showed a very robust change in the mKikume red/green fluorescence ratio over time for dividing, but not for non-dividing bacteria (Fig. 1c,d). Furthermore, flow cytometry analysis of exponentially growing *S*. *aureus* showed that complete recovery from photoconversion occurred within 90 min (Fig. 1e,g).

**Figure 2:**
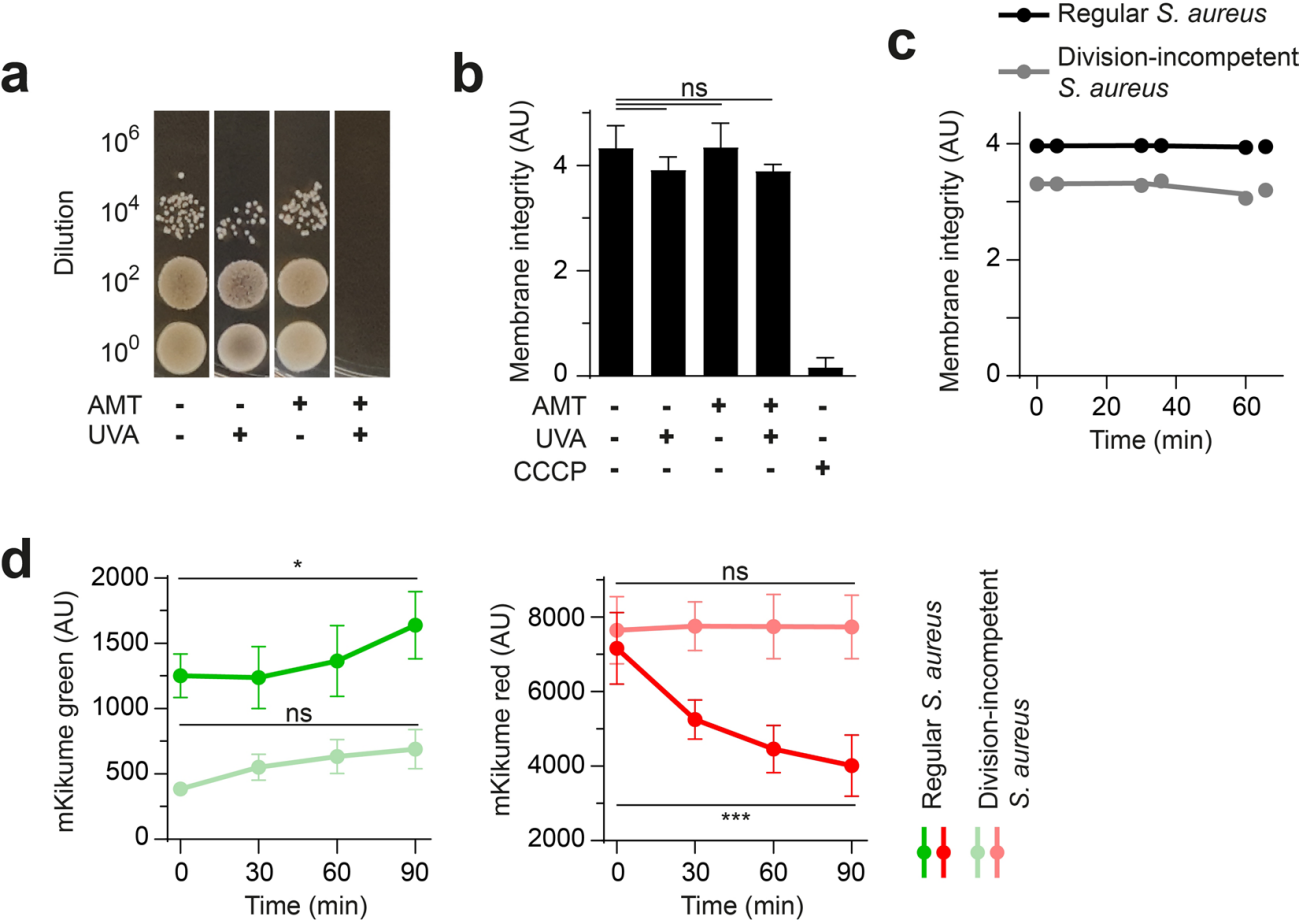
Recovery from photoconversion shows mainly proliferation, not protein turnover. (**a**) Efficiency of growth inhibition after generation of division-incompetent, but metabolically active bacteria. Serial dilutions of control bacteria and bacteria treated with 10 µM psoralen (AMT), or 10 min 375 nm (UVA), or both. (**b**) Flow cytometry measurement of membrane integrity by using DiOC_2_ of treated bacteria with 10 µM psoralen (AMT) and 10 min 375 nm (UVA). CCCP was used as a low membrane integrity control. Mean of four individual cultures + standard deviation is shown; ns, not significant as determined by one-way ANOVA. (**c**) Membrane integrity of division-incompetent, but metabolically active bacteria (grey) and control *S*. *aureus*-pKikume (black) over time in culture. Each symbol represents one individual culture, the curves connect the mean. (**d**) mKikume red and green fluorescence development over time. Measurement of fluorescent bacteria by flow cytometry, started after photoconversion of exponential growth (red, green) and division-incompetent (pale red and green) bacteria. Mean +/− standard deviation of 5–7 individual cultures per condition is shown. ^***^p < 0.001; ^*^p < 0.05 as determined by two-way ANOVA.

Recovery from photoconversion could, in principle, also occur through fluorescence protein turnover in non-dividing bacteria. In order to address the contribution of such a growth-independent impact on biosensor readout, we employed division-incompetent, but metabolically active *S*. *aureus* generated using psoralen and UV DNA crosslinking^20,21^. These bacteria were completely non-proliferative, but unaffected in their membrane potential (Fig. 2a,c). However, for the division-incompetent *S*. aureus-pKikume, we observed a significant reduction in recovery from photoconversion as compared to controls (Fig. 2d). Therefore, indeed bacterial proliferation, and not protein turnover, is mainly responsible for the fluorescence recovery readout of the biosensor.

### Longitudinal *S*. *aureus* growth measurement *in vivo* shows maximal proliferation dampening upon the arrival of neutrophils at the site of infection

**Figure 3:**
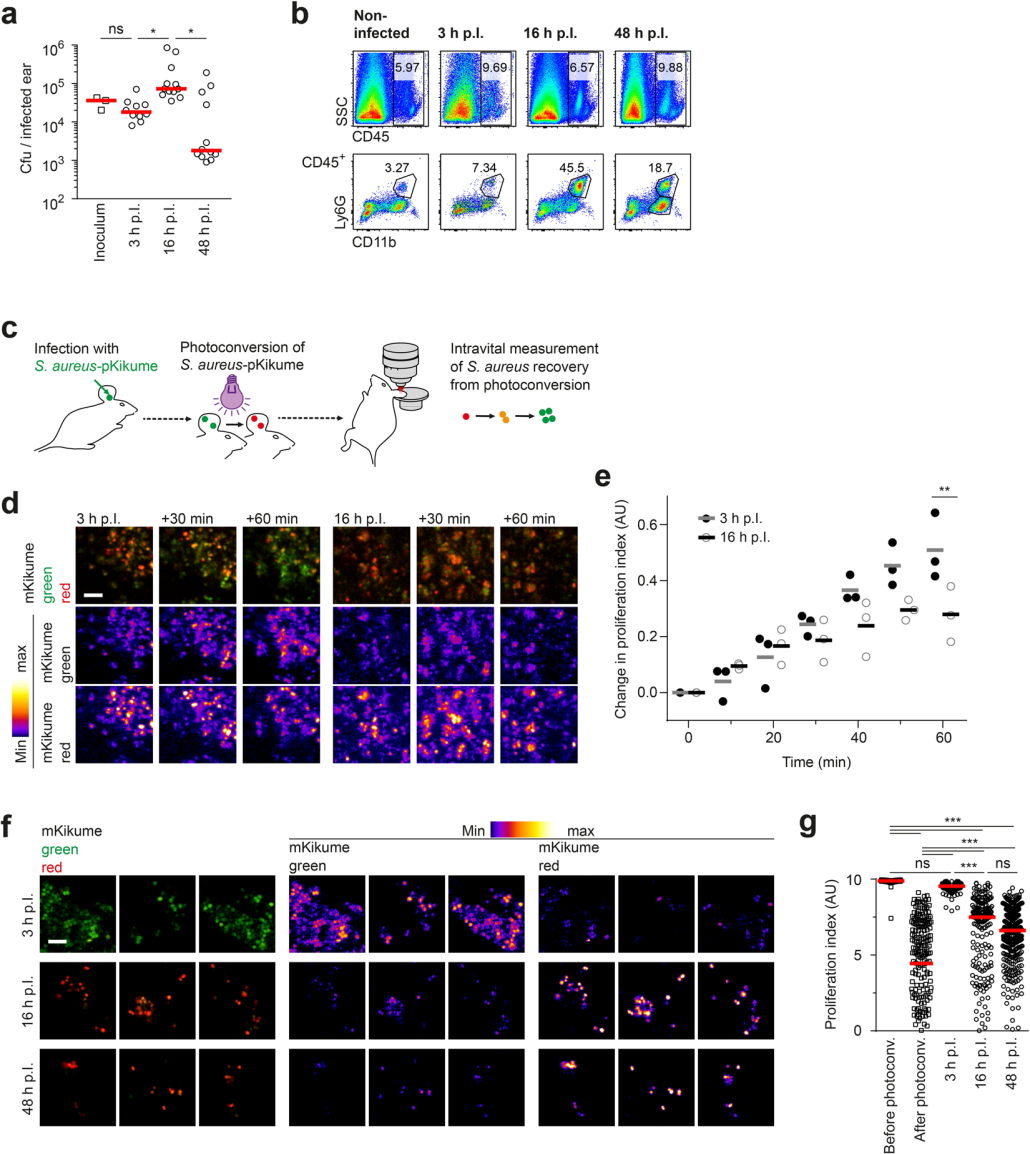
*S*. *aureus* proliferation decreases after the onset of the innate immune response. (**a**) Pathogen burden at 3, 16, and 48 h p.I. in *S*. *aureus*-pKikume infected mice. Each symbol represents one individual ear, the inoculum is shown for comparison. ^*^p < 0.05 as determined by one-way ANOVA. (**b**) Flow cytometry of leukocytes recruited to the site of *S*. *aureus*-pKikume infection at 3, 16, and 48 h p.I. Data representative of eight infected ears per condition. (**c**) Schematic representation of the intravital 2-photon growth measurement experiment. After a defined period of time p.I., bacteria at the infection site were photoconverted and the mKikume red and green fluorescence was measured over time by intravital 2-photon microscopy. (**d**) Examples of intravital 2-photon microscopy of infected mouse ears starting after photoconversion 3 h (left panel) versus 16 h (right panel) p.I. Three-dimensional projections of 20 Z-slices spaced 2 µm are shown. The single red and green fluorescence channels in the middle and bottom rows of each panel are shown as heat maps. Scalebar, 10 µm. (**e**) Proliferation index measurement by intravital 2-photon microscopy over time at 3 h p.I. (closed symbols) versus 16 h p.I. (open symbols). Representation of the 80^th^ percentile changes in 10 min intervals of three imaged regions. Each dot indicates a 10 min interval of one region analysed. Horizontal bars represent the mean. ^**^p < 0.01; as determined by two-way ANOVA. (**f**) Confocal imaging of photoconverted *S*. *aureus*-pKikume in fixed cryosections of ears infected for the indicated times, photoconverted 1 h prior to fixation and analysis. Four representative regions per condition are shown, the single red and green fluorescence channels in the middle and right column are shown as heat maps. Scale bar, 5 µm. (**g**) Proliferation index of bacteria in fixed cryosections of ears infected for 3, 16, and 48 h, photoconverted 1 h prior to fixation and analysis. Confocal images as represented in (**f**) were analysed automatically. At least 10 confocal images per mouse ear were analyzed in at least five ears per time point. Each symbol represents one confocal image; horizontal bars represent the median; ^***^p < 0.001; ns, not significant as determined by one-way ANOVA.

Neutrophils represent the first line of cellular defenses against *S*. *aureus* infection. Upon recruitment from the bloodstream, these professional phagocytes are crucial for the containment and degradation of microorganisms at the site of infection^5^. Consequently, while *S*. *aureus* burden in the tissue increased significantly between 3 and 16 h p.I., bacterial numbers had dropped below initial numbers by 48 h p.I. (Fig. 3a). This was in line with a significant recruitment of CD45^+^ leukocytes at the site of infection at 16 h p.I., which mainly consisted of Ly6G^+^CD11b^+^ neutrophils (Fig. 3b). The decline of pathogen numbers could be in principle achieved by killing of a constantly growing bacterial population, or, alternatively, by also inhibiting the individual bacterial growth rates. In order to analyze whether *S*. *aureus* growth rate was changed upon the massive neutrophil recruitment, we compared bacterial recovery from photoconversion at 3 versus 16 h p.I. by intravital 2-photon microscopy. To this end, B6 albino mice were infected intradermally in the ear with *S*. *aureus*-pKikume. Control immunofluorescence staining of *S*. *aureus* in fixed ear tissues showed that all bacteria at the site of infection expressed the mKikume protein and would thus be detectable during intravital 2-photon microscopy (Supplementary Fig. S2). Bacteria at the site of infection were photoconverted by 405 nm illumination at 3 h post infection (p.I.) and immediately imaged by intravital 2-photon microscopy for subsequent 60 min (Fig. 3c). Similar to the observations in the *in vitro* system, substantial increase in green and decrease in red mKikume fluorescence was detected after photoconversion (Supplementary Fig. S3a, Supplementary movie 1). Importantly, the mKikume red/green fluorescence ratio and consequently a proliferation index calculated from this ratio, reached a plateau within 60 min (Supplementary Fig. S3b,c), which was comparable with the kinetics observed *in vitro* (see Fig. 1f,g). This suggests that 3 h p.I., *S*. *aureus* proliferation in the tissue can reach rates that are comparable to exponential growth *in vitro*. Strikingly, we observed a significant decline in the rate of recovery from photoconversion at 16 h p.I. compared to 3 h p.I. (Fig. 3d,e, Supplementary movie 2). Of note, recovery from photoconversion was not completely abrogated at 16 h p.I. Thus, we concluded that at 16 h p.I., most of the observed *S*. *aureus* remained alive initially, but exhibited a reduced growth as compared to 3 h p.I. As the bacterial numbers at 48 h p.I. were nearly 100 times lower than at 16 h p.I. and thus complicated a robust determination of proliferation rates from intravital 2-photon microscopy, we devised a quantitative confocal microscopy approach based on cryosections of *S*. *aureus*-pKikume infected ears fixed 60 min after photoconversion. Automated determination of mKikume red and green fluorescence of these samples allowed for measuring proliferation rates in a large number of tissue sections in a standardized fashion. Confirming our intravital 2-photon microscopy observations, proliferation rates determined from automated analysis of tissue sections were dramatically lower at 16 h versus 3 h p.I., but did not significantly deline further at 48 h p.I. (Fig. 3f,g). Thus, concomitantly with the onset of the innate immune reaction, the growth rate of the *S*. *aureus* at the site of infection rapidly decreases.

### NADPH oxidase activity contributes to the dampening of *S*. *aureus* growth

**Figure 4:**
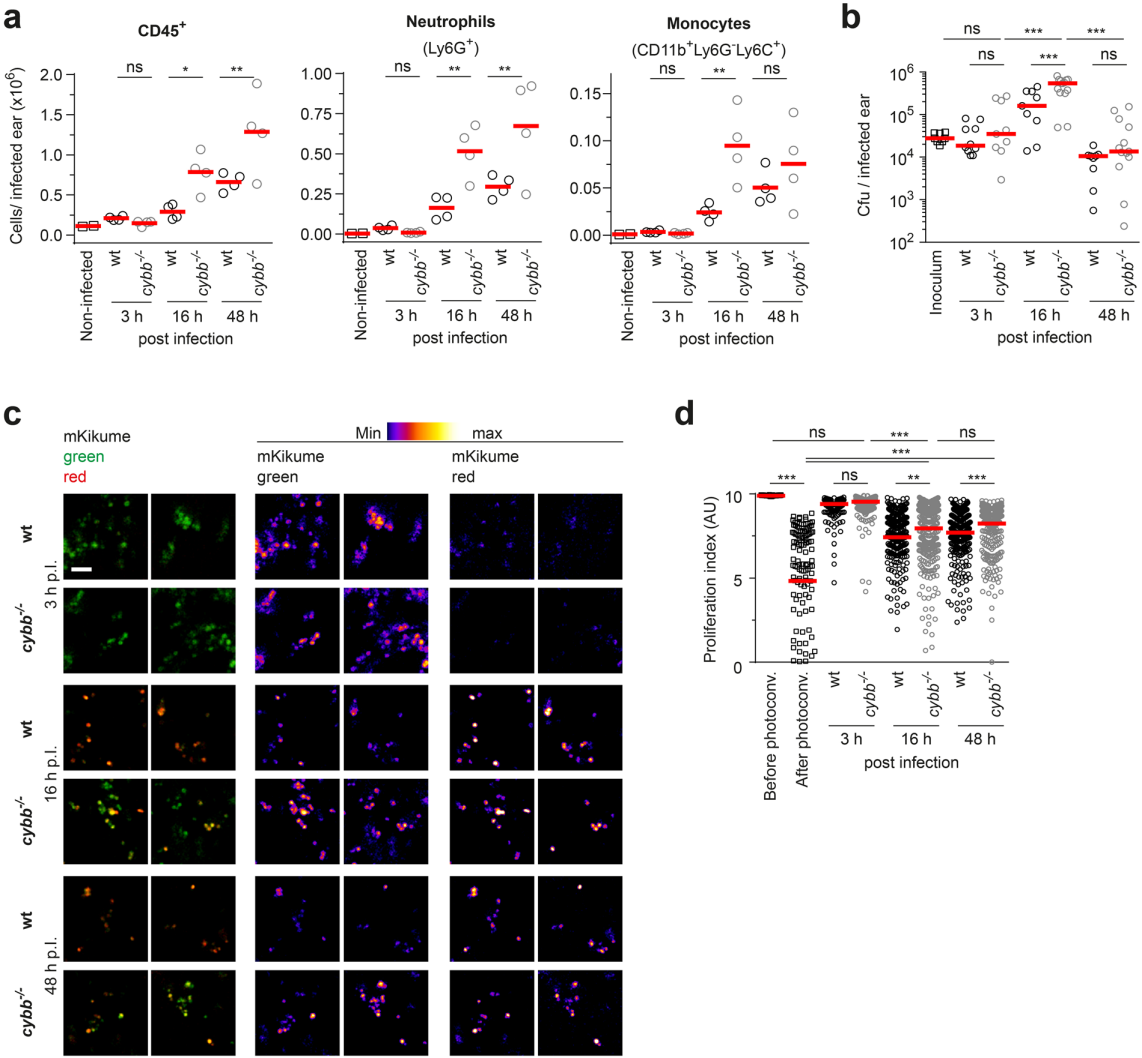
Oxidative burst reduces *S*. *aureus* growth rates during control of the infection. (**a**) Cells counts in infected ears analysed by flow cytometry. Gating on CD45^+^ cells, Neutrophils (CD11b^+^, Ly6G^+^) and Monocytes (CD11b^+^, Ly6G^−^, Ly6C^+^) as shown in Supplementary Fig. S4 (**a**). Each dot represents one individual ear; horizontal bars represent the mean; ^**^p < 0.01; ^*^p < 0.05; ns, not significant as determined by one-way ANOVA. (**b**) Pathogen burden at 3, 16, and 48 h p.I. in wt (black symbols) and *cybb*^*−/−*^ (grey symbols) mice infected with *S*. *aureus*-pKikume. Each symbol represents one individual ear, plating of the inoculum is shown for comparison; horizontal bars represent the median; ^***^p < 0.001; ns, not significant as determined by one-way ANOVA. (**c**) Confocal imaging of photoconverted *S*. *aureus*-pKikume in fixed cryosections of wt and *cybb*^*−/−*^ mice infected for 3, 16, and 48 h, photoconverted 1 h prior to fixation and analysis. Two representative regions are shown per condition, the single red and green fluorescence channels in the middle and right column are shown as heat maps. Scale bar, 5 µm. (**d**) Proliferation index of bacteria detected in in fixed cryosections as shown in (**c**) of ears infected and analysed automatically. At least 10 confocal images per mouse ear were analyzed in four to 10 ears per condition. For controls three ears per condition were analyzed. Each dot represents one confocal image; horizontal bars represent the median; ^***^p < 0.001; ^**^p < 0.01; ns, not significant as determined by one-way ANOVA.

The production of reactive oxygen by NADPH oxidase constitutes a major antimicrobial effector mechanism of neutrophils. In addition to its importance for oxidative burst acting directly against phagocytosed microorganisms, superoxides produced by this enzyme can trigger the release of NETs^7,8^. Whether NADPH oxidase activity can impair pathogen proliferation rates or mediate direct killing of *S*. *aureus* without overt impact on its proliferation has remained completely unknown. We therefore set out to analyze *S*. *aureus* proliferation rates in NADPH oxidase-deficient *cybb*^−/−^ mice. We observed an increased recruitment of both neutrophils and monocytes to the infected ears of *cybb*^−/−^ animals, suggesting that NADPH oxidase-deficiency resulted in a loss of pathogen containment and increased inflammation (Fig. 4a, Supplementary Fig. S4). Also, at 16 h. p.I., bacterial burden was found to be significantly increased in *cybb*^*−/−*^ animals (Fig. 4b). Strikingly, analysis using the proliferation biosensor revealed a significantly higher *S*. *aureus* growth rates in *cybb*^−/−^ mice compared to wildtype both at 16 h and 48 h, but not at 3 h p.I. (Fig. 4c,d). Of note, the bacterial growth rates were not completely rescued to the levels found at 3 h p.I. in the *cybb*^*−/−*^ mice, suggesting that besides reactive oxygen production, other mechanisms limit bacterial growth, which could also explain that ultimately, the infection is controlled in both experimental groups in our cutaneous infection model (see Fig. 4b). Nevertheless, our data show that NADPH oxidase contributes to the reduction of bacterial numbers by dampening *S*. *aureus* growth.

### *S*. *aureus* is located within neutrophils at time points of efficient confinement

**Figure 5:**
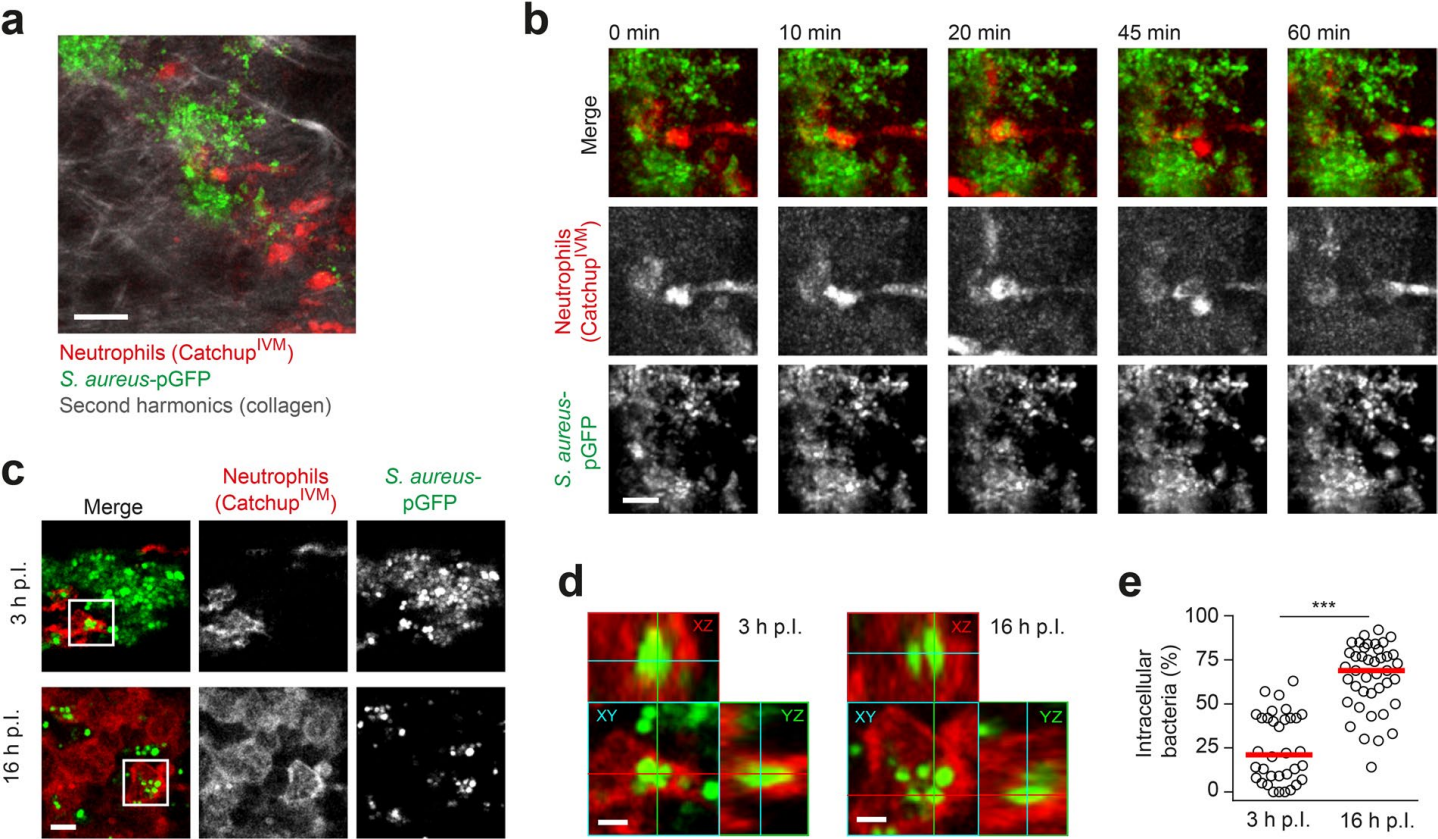
*S*. *aureus* is located within neutrophils at time points of efficient confinement. (**a**) Overview of intravital 2-photon microscopy of Catchup^IVM^ neutrophil reporter mice infected with GFP-expressing *S*. *aureus*-pGFP. Projection of three-dimensional image of 11 Z-slices spaced 3 µm is shown. Scale bar, 20 µm. (**b**) Time-lapse intravital 2-photon microscopy of a neutrophil (red) taking up *S*. *aureus* (green) 4 h p.I. Projections of three-dimensional images of 11 Z-slices spaced 3 µm are shown. Scale bar, 10 µm. (**c**) Cryosections from Catchup^IVM^ neutrophil reporter mice infected with *S*. *aureus*-pGFP in the ear for 3 and 16 h. Scale bar, 5 µm. (**d**) 3D-representation of bacteria (green) within neutrophils (red) shown in c). Scale bar, 2 µm. (**e**) Bacteria and neutrophils were automatically 3D-segmented and bacteria were assigned either inside or outside the detected neutrophil shapes. At least five 3D-segmented volumes (120 × 120 × 20 µm^3^) per infected ear in four to five ears per condition were analysed and the percentage of bacteria localized in neutrophils was calculated for each volume. Each dot represents one confocal volume analysed; horizontal bars represent the median; ^***^p < 0.001 as determined by Mann-Whitney test.

Neutrophil NADPH oxidase can be involved in control of both extracellular bacteria via NETs^8^, as well as intracellular bacteria via oxidative damage^7,22^. In order to visualize the interaction of neutrophils with *S*. *aureus* upon recruitment to the site of infection and to analyze bacterial localization, we employed Catchup^IVM^ mice, in which neutrophils exhibit red fluorescence dependently of neutrophil-specific Ly6G expression^23^. When we infected these mice with GFP-expressing *S*. *aureus* (*S*. *aureus*-pGFP), we observed using intravital 2-photon microscopy 3 h p.I. the uptake of bacteria by neutrophils (Fig. 5a,b, Supplementary movie 3). Interestingly, quantitative analysis of *S*. *aureus* cellular localization revealed that while the small number of neutrophils present at 3 h p.I. had taken up a fraction of the bacteria, the majority of *S*. *aureus* was found within these cells at 16 h p.I. (Fig. 5c–e). Thus, we conclude that *S*. *aureus* is internalized by recruited neutrophils right after the onset of the innate immune response and bacterial growth is mainly controlled intracellularly. The intracellular environment in *cybb*^*−/−*^ versus wildtype host cells could differentially impact on the proliferation biosensor readout instead of proliferation itself. To exclude this possibility, we compared the recovery from photoconversion of untreated versus division-incompetent *S*. *aureus* after uptake into bone marrow-isolated neutrophils from *cybb*^*−/−*^ and wildtype mice. *S*. *aureus* which were division-incompetent (see Fig. 2) showed substantially less recovery from photoconversion both in *cybb*^*−/−*^ as well as wildtype neutrophils, indicating that proliferation measurement *per se* is not affected by the presence or absence of NADPH oxidase (Supplementary Fig. 5a). In line with our observations *in vivo* (see Fig. 4), we could observe that dilution of mKikume red fluorescence in *cybb*^*−/−*^ neutrophils is slightly enhanced in comparison to both wildtype neutrophil infection as well as division-incompetent *S*. *aureus* (Supplementary Fig. 5b). Thus, also *in vitro*, *cybb*^*−/−*^ neutrophils might be permissive for *S*. *aureus* division.

Therefore in summary, our data suggest that NADPH oxidase-mediated dampening of *S*. *aureus* growth rates upon uptake into neutrophils contributes to control of the infection.

## Discussion

The emergence and spread of antibiotic-resistant *S*. *aureus* demands a better understanding of the persistence mechanisms, lifestyle and physiological state of *S*. *aureus* at sites of infection. The bacterial growth rate is a fundamentally important parameter for elucidating these questions, but has remained difficult to measure so far. Determination of the bacterial burden via colony forming units is insufficient for this purpose, as it cannot distinguish whether pathogen numbers are controlled via killing of the bacteria, or reduction of the proliferation rate, which in principle can be non-lethal for the microbes. To circumvent this, dilution-based approaches have been applied for measuring the proliferation of the bacteria shortly after infection, e.g. by pulsing *S*. *aureus* with fluorescent dyes^16,24,25^, or with chemical inducers that trigger a short peak of fluorescence protein production^14^. However, this limits the window of measurement to only few division cycles after infection, or to the homogenous accessibility of the tissue of interest for the inducer, respectively. Protein maturation-dependent fluorescent timers have been introduced as a pulse-free alternative for measuring the *de novo* production of bacterial protein, but are strongly influenced by the oxidative state of the microenvironment^26^. As this approach is not based on the fluorescence protein levels, but maturation kinetics, its use for the investigation of innate immune defense mechanisms is complicated^27^.

We show that a photoconversion-based biosensor can be used for probing bacterial growth in a non-invasive fashion. Very much in contrast to alternative fluorescence dilution-based approaches, our system allowed bacterial proliferation measurement at any time point during the ongoing infection, even days after inoculation. This has enabled us to dissect for the first time the impact of NADPH oxidase on *S*. *aureus* growth rates *in vivo* not only before and after neutrophil recruitment, but also at later phases when the pathogen is about to be cleared. Of note, the arrival of neutrophils and phagocytosis of the *S*. *aureus* coincided with a reduction of bacterial growth. Importantly however, we show a slight recovery from photoconversion of *S*. *aureus* also at 16 h p.I., when the vast majority of the bacteria is located within recruited neutrophils. It is possible that such a residual proliferative activity is limited to the small population of bacteria not locates within neutrophils at this time point. However, the observation is interesting with regard to *in vitro* data suggesting that *S*. *aureus* growth is not completely abolished within professional phagocytes. For example, bacterial proliferation has been shown in the lysosomal compartment of macrophages^15^ and dendritic cells^28^. Similarly, it was shown that the bacteria can grow within Kupffer cells in the liver after a systemic infection^16^. For neutrophils, intracellular survival has been shown, however direct evidence for intracellular replication is still lacking^10^. Of note, we show that compared to the mainly extracellular bacteria at 3 h p.I., pathogen growth is substantially reduced at phases at which the *S*. *aureus* have been phagocytosed by neutrophils. The situation could be different in NADPH oxidase deficient neutrophils: Although we found that *S*. *aureus* burden was significantly elevated in NADPH oxidase deficient mice after, but not before neutrophil recruitment to the site of infection, the strong reduction in the proliferation of *S*. *aureus* after neutrophil recruitment was partially rescued under NADPH oxidase deficiency. Therefore, we conclude that the recruited phagocytic cells rely on NADPH oxidase not only to directly kill *S*. *aureus*, but also to restrict bacterial growth non-lethally. At time points at which NADPH oxidase deficiency results in elevated pathogen burden, most *S*. *aureus* are located within neutrophils. This suggests that the observed contribution of ROS production to the inhibition of bacterial growth is taking effect within the recruited phagocytes after uptake of the pathogen.

Taken together, our proliferation reporter system enables us to dissect non-invasively, and throughout the whole course of the infection in the tissue, the impact of the neutrophil response on *S*. *aureus in vivo*. This approach has the potential of providing a better understanding of the interplay between host immune defenses and the physiological adaptations of the pathogen during infection.

## Methods

### Mice

Age- and sex-matched wild-type C57BL/6J and *cybb*^−/−^ (B6.129S-*Cybb*^*tm1Din*^/J) mice were bought from Jackson Laboratories (Bar Harbor, MA), B6 albino (B6N-*Tyr*^*c-Brd*^/BrdCrCrl) mice were purchased from Charles River (Sulzfeld, Germany), and Catchup^IVM^ mice were obtained by crossing C57BL/6-*Ly6g*^*tm2621(Cre-tdTomato)Arte*23^ mice to B6.Cg-*Gt(ROSA)26Sor*^*tm14(CAG-tdTomato)Hze*^ mice (kindly provided by Monika Riek-Burchardt, Otto-von-Guericke-University Magdeburg). All mice were bred under particular pathogen-free conditions at Otto-von-Guericke-University, Magdeburg.

All animal experiments were reviewed and approved by the Ethics Committee of the Office for Veterinary Affairs of the State of Saxony-Anhalt, Germany (Permit License Number 42502-2-1314 Uni MD) in accordance with legislation of both the European Union (Council Directive 499 2010/63/EU) and the Federal Republic of Germany (according to § 8, Section 1 TierSchG, and TierSchVersV).

### Bacterial strains, media and cultivation

Bacteria were cultivated in Brain Heart Infusion Broth (Carl Roth) at 37 °C with shaking or on 1.5%-agar plates (Carl Roth), supplemented with 12.5 µg/mL chloramphenicol (Roth). For OD_600_ measurement BHI medium was used as reference and the culture was diluted with BHI medium to obtain values between 0.01 and 1.0. For calculations OD_600_ = 1 was assumed to correspond to 10^8^ bacteria/ml.

### Generation of division-incompetent, but metabolically active bacteria

To generate division-incompetent, but metabolically active bacteria, pyrimidine bases of the DNA were crosslinked by psoralen with long-wavelength UVA light^20,21^. For this, 10 µM of 4′-(aminomethyl)-4,5′,8-trimethylpsoralen hydrochloride (AMT, Sigma Aldrich) were added to the S. aureus culture. 0.5 ml of this sample were illuminated in a petri dish on ice with violet light at 357 nm wavelength using an assembly of 3 × 3 LED diodes (Strato, half-viewing angle: 10°; Radiant Power: 10 mW) for 10 min in a distance of 0.8 cm.

### Measurement of membrane integrity

To measure the viability of the bacteria, the BacLight^TM^ Bacterial Membrane Potential Kit (Molecular Probes, Invitrogen) was used according to manufacturer’s instructions and measured by flow cytometry. As control for intact membrane potential, a *S*. *aureus* day culture was used in which the membrane potential was abolished using the proton ionophore carbonyl cyanide 3-chlorophenylhydrazone (CCCP). As indicator, 3,3′- Diethyloxa-carbocyanine iodide (DiOC_2_) was used, in which green fluorescence (measured at 488 nm excitation and 530/30 nm emission) shifts to red fluorescence (measured at 488 nm excitation and 670/30 nm emission) by self-association in high cytosolic concentrations.

### *S*. *aureus* infection

A day culture was inoculated by an overnight culture (16–18 h) to an OD_600_ 0.04–0.06. The bacteria are harvested for infection at OD_600_ 0.4–0.6, which represented the early exponential growing phase. After washing with cold PBS, the OD_600_ was adjusted to 2.5 in PBS by centrifugation (9000 g, 5 min, 4 °C) and resuspension. The ear skin of mice was intradermally infected at three sites per ear, each with 5 × 10^4^ bacteria using a 35 gauge syringe.

### Determination of bacterial tissue burden

To analyze colony-forming units in the ear, bacteria were isolated from infected ears by homogenization with 15 mL disposable tissue grinders (Fisherbrand) or 15 mL tapered tissue grinders (Wheaton). The cells were centrifuged (16500 g, 3 min, 4 °C) and lysed with 1% triton-X 100 (Roth) in H_2_O. The bacteria were serially diluted in triplicates and plated on agar plates.

### Photoconversion

For *in vitro* photoconversion, *S*. *aureus*-pKikume were illuminated in a 96-well plate with violet light at 405 nm wavelength using an assembly of 2 × 2 LED diodes (Strato, half-viewing angle: 15°; Radiant Power: 10 mW) for 1 minute in a distance of 1.7 cm. In Fig. 1a, the *in vitro* photoconversion of *S*. *aureus*-pKikume was done by illumination in a petri dish with violet light at 357 nm wavelength by assembling 3 × 3 LED diodes (Strato, half-viewing angle: 10°; Radiant Power: 10 mW) for 5 minutes in a distance of 0.8 cm.

Ears of anaesthetized mice were photoconverted with violet light at 405 nm wavelength using an assembly of 3 × 3 LED diodes (Strato, half-viewing angle: 15°; Radiant Power: 10 mW), for intravital 2-photon microscopy illumination tuime was set to 1–2 minutes in a distance of 2 cm, for confocal microscopy, the ears were illuminated from each side for 1 minute in a distance of 1.5 cm

### Flow cytometry

Flow cytometry of bacteria was performed in culture medium or diluted to suitable concentrations with cold PBS. The red mKikume fluorescence signal was measured at 488 nm excitation and 610/20 nm emission, the green mKikume fluorescence signal was measured at 488 nm excitation and 530/30 nm emission.

For analysis of cells recruited to the site of infection, ears were separated into dorsal and ventral sheets using forceps and digested with 1 mg/mL collagenase (Sigma), 50 ng/mL DNase (Sigma-Aldrich), 100 µg/mL penicillin/streptomycin (Biochrom) and 5 µg/mL tetracycline (Roth) in RPMI 1640 Medium (Biochrom) at 37 °C and 600 rpm shaking for 60 minutes. To release the cells the ear were homogenized through a 70 µm cell strainer (Falcon) and washed with PBS. After centrifugation for 10 min with 1500 rpm at 4 °C and washing with Buffer (0.5% FCS (PAA); 2 mM EDTA, (Carl Roth); in PBS (Biochrom)), the cells were Fc-blocked using anti-CD16/32 antibody (clone 93) (BioLegend), and stained with PerCp-Cy5.5 conjugated anti-CD45 (clone 30-F11), APC-Cy7 conjugated anti-CD11b (clone M1/70), PE conjugated anti-CD11c (clone N418), APC conjugated anti-Ly6G (clone 1A8), PE-Cy7 conjugated anti-Ly6C (clone HK1.4) (all from Biolegend). CountBright^TM^ absolute counting beads (Invitrogen) were added to the samples before measurement. The measurement was performed with a LSRFortessa flow cytometer (BD Biosciences) using the blue 488 nm and the red 640 nm lasers. The data were analyzed by using the FlowJo X software (FlowJo, LLC).

### Intravital two-photon microscopy

The mice were anesthetized and prepared for intravital two-photon imaging. The mouse was placed on a heating stage adjusted to 37 °C and the ear was fixed to a metal platform. A coverslip sealed to a surrounding parafilm blanket was placed onto the ear and glued to the platform. Two-photon imaging was performed Zeiss LSM 700 equipped with a Mai Tai DeepSee Ti:Sa laser (Spectra-Physics) tuned at 920 nm (*S*. *aureus*-pKikume in B6 albino mice) or 960 nm (*S*. *aureus*-GFP in Catchup^IVM^ mice) and a W Plan-Apochromat 20x/1.0 DIC VIS-IR dipping objective (Zeiss). For *S*. *aureus*-pKikume, the emitted signal was split by 490 nm, 520 nm and 555 nm long pass and dichroic mirrors and filtered with 509/22 nm, 500/50 nm (green mKikume) and 589/54 nm (red mKikume) filters before collection with non-descanned detectors. For *S*. *aureus*-GFP in Catchup^IVM^ mice the emitted signal was splitted by 555 nm long pass and 490 nm long pass and dichroic mirrors and filtered with 565/10 nm (red signal, neutophils), 485 nm short pass (second harmonics) and 500/50 nm (GFP signal) filters before collection with non-descanned detectors.

### Confocal microscopy

For *in vitro* imaging of *S*. *aureus* proliferation, agar pads (2% low melting agarose (Serva), 10% FCS (PAA), 12,5 µg/mL chloramphenicol (Roth) in RPMI medium 1640, without phenol red (gibco) on glass bottom dishes (ibidi) were used. For analyzing the bacteria in the tissue, the infected ears were harvested at defined time points, fixed for 8–16 h in 4% paraformaldehyde (Roth) in PBS (Biochrom) at 4 °C. Afterwards the fluorescence was stabilized by incubating the ears for 8–16 h in 20% sucrose (Carl Roth) at 4 °C. The samples were frozen in Tissue-Tek® O.C.T.™ Compound (Sakura) with liquid nitrogen and stored at −80 °C. The ears were cut into 20 µm cryosections and placed on Superfrost slides (Thermo Scientific) coated with 0.1% Poly-L-Lysin (Sigma-Aldrich) in H_2_O and air-dried for 1–2 h before storage at −40 °C until use.

Measurement was performed with the TCS SP8 confocal laser scanning microscope (Leica Microsystems) using a 63x/1.40 Oil CS2 objective. For proliferation analysis the red mKikume fluorescence signal was measured at 561 nm excitation and 598–638 nm emission, while the green mKikume fluorescence signal was measured at 488 nm excitation and 526–570 nm emission.

To extract data of *S*. *aureus* uptake by neutrophils, the red tdTomato fluorescence signal (neutrophils in the Catchup^IVM^ mice) was measured at 561 nm excitation and 600–669 nm emission, while the green GFP fluorescence signal (*S*. *aureus*-pGFP) was measured at 488 nm excitation and 499–565 nm emission.

### Image analysis

Bacteria in intravital 2-photon microscopy movies were segmented using the surface detection function of the Imaris software (Bitplane) from combined red and green mKikume fluorescence channels. Fluorescence values and time points of the segmented bacteria for the individual channels were converted into flow cytometry datasets using the DiscIT software^29^ and analyzed using the FlowJo X software (FlowJo, LLC).

For analysis of confocal microscopy data, 144 × 144 μm frames spaced at least 2.5 μm apart were used to detect bacteria in a combined green and red mKikume channel using the threshold and analyze particle functions of the Fiji software (NIH, http://rsb.info.nih.gov/ij/). Fluorescence data from the resulting regions of interest were extracted, converted into flow cytometry datasets using the DiscIT software^29^ and analyzed using the FlowJo X software (FlowJo, LLC). The combined proliferation indices of all detected bacteria within one frame were used for data representation.

## Supplementary information

Supplementary material

Supplementary Movie 1

Supplementary Movie 2

Supplementary Movie 3

## Data Availability

All relevant data shown included in the manuscript and supplemental material, raw data are available without restriction from the corresponding author upon request.
