# Supplementary material for: Longitudinal proliferation mapping in vivo reveals NADPH oxidase-mediated dampening of Staphylococcus aureus growth rates within neutrophils
Source: Sci Rep. 2019 Apr 5;9:5703. doi: 10.1038/s41598-019-42129-6 (PMC6450975; doi:10.1038/s41598-019-42129-6)
Supplement: Supplementary file 1 — Supplementary material [file 41598_2019_42129_MOESM1_ESM.pdf]

Supplementary information

Longitudinal proliferation mapping *in vivo* reveals NADPH oxidase-mediated dampening of *Staphylococcus aureus* growth rates within neutrophils

Elena A. Seiß*, Anna Krone, Pauline Formaglio, Oliver Goldmann, Susanne Engelmann, Burkhart Schraven, Eva Medina, Andreas J. Müller*

* Corresponding authors: Email: elena.seiss@med.ovgu.de, andreas.mueller@med.ovgu.de

Supplementary methods

Supplementary movie legends

Supplementary figure S1. An *in vivo* proliferation biosensor system for *S. aureus*.

Supplementary figure S2. Sensitivity of mKikume fluorescence for detecting *S. aureus* in the tissue.

Supplementary figure S3. Tracking *S. aureus* proliferation in the ongoing infection by intravital 2-photon microscopy.

Supplementary figure S4. Recruitment of immune cells in *cybb*$^{-/-}$ mice.

Supplementary figure S5. NADPH oxidase-mediated dampening of *S. aureus* recovery from photoconversion in *in vitro*-infected neutrophils.

Supplementary Methods

Bacteria strains

The gene encoding mKikume[1] was codon optimized for *S. aureus*, *de novo* synthetized (Eurofins MWG) and inserted using ApaI and EcoRI into the pGL485 plasmid[2] yielding the plasmid pLacKikume. The truncated *lacI* gene including *pcn* promoter in front of the mKikume were exchanged via ApaI and SalI restriction sited by a construct comprising the *sarA* P1 promotor, *sod* RBS[3] and iTag[4] (*de novo* synthetized), resulting in the plasmid pKikume. To generate the pTufAKikume, a construct including the *tufA* promoter and ribosome binding site spanning 300bp upstream of the *tufA* cds and an iTag (*de novo* synthetized) were cloned into the pKikume plasmid via ApaI and SalI. The recombinant plasmids were introduced into the *S. aureus* strain RN4220[5] by electroporation (settings: 2.0-2.5 kV, 100 Ω, 25 µFd, time constant= 2.54-2.60 ms, Gene Pulser II System BIORAD). The transfer of the plasmids into the *S. aureus* SH1000[6] was done by phage transduction with bacteriophage 85[7]. GFP-expressing *S. aureus* pGFP have been described previously[2].

For blood agar, 10% defibrinated sheep blood (Thermo Scientific) was added to LB agar (Carl Roth). *E. coli* DH5α was cultivated in LB medium (Carl Roth) at 37°C with shaking or on LB-agar plates (Carl Roth), both containing 100 µg/mL spectinomycin.

Wide-field microscopy

To test the photoconvertability, a wide-field microscope was used. Fluorescence signals were acquired by a BX61 microscope (Olympus) equipped with a UPlanApo 40x/0.85 objective (Olympus) and a F-view camera (Olympus). Bacteria from a day culture (OD_{600}=0.4-0.6) were pipetted on an agar pad (2% low melting agarose (Serva), 10% FCS (PAA), 12,5 µg/mL chloramphenicol (Roth) in RPMI medium 1640 without phenol red (gibco) and placed on an coverslip. Photoconversion was performed for 20 seconds with 436/10 nm by half power of the illumination system MT_{20} (Olympus).

Staining of tissue sections for confocal microscopy

To for immunofluorescence staining of the bacteria, the infected ears (4 h p.I.) were prepared as for confocal microscopy. After storage at -40 °C, the tissue was permeabilized with 0.1 % triton-X 100 (Roth) in PBS and stained with polyclonal rabbit anti-*Staphylococcus aureus* (from abcam) and Alexa Fluor 647 conjugated goat anti-rabbit IgG (Jackson ImmunoResearch). The fluorescence signal of the antibody staining was measured at 488 nm excitation and 499-525 nm emission, while the green mKikume fluorescence signal was measured at 633 nm excitation and 680-750 nm emission.

In vitro cell culture infections and analysis

Neutrophils were purified from the bone marrow of wildtype and *cybb*$^{-/-}$ mice by magnetic-activated cell sorting as described previously[8]. The neutrophils were infected in RPMI 1640 medium (Merck) containing 10% fetal bovine serum (Pan Biotec GmbH) at a multiplicity of infection of 10:1 with division incompetent or control *S. aureus*-pKikume (see main methods). After 30 minutes, the cell culture was photoconverted (see main methods), extracellular bacteria were killed by addition of Lysostaphin (Sigma-Aldrich, 2.5 µg/ml final concentration for 10 minutes), washed with RPMI 1640 medium containing 10% fetal bovine serum, and cultivated for 50 minutes at 37°C 5% CO_2. After this, cells were put on ice and stained with APC conjugated anti-CD11b (clone M1/70), and BV421-conjugated anti-Ly6G (clone 1A8) antibody (Biolegend). The measurement was performed with a LSRFortessa flow cytometer (BD Biosciences) using the violet 405 nm, blue 488 nm, green 561 and the red 640 nm lasers.

Supplementary movie legends

Supplementary movie 1

Three examples of recovery from photoconversion in the ongoing infection as shown in Figure 2 b. Intravital 2-photon microscopy of *S. aureus*-pKikume infected mouse ears starting after photoconversion 3 h p.I.. Scale bar, 10 µm.

Supplementary movie 2

Differential recovery from photoconversion at early and late time points p.I. as shown in Figure 3 d. Intravital 2-photon microscopy of *S. aureus*-pKikume infected mouse ears starting after photoconversion 3 h (left) versus 16 h (right) p.I.. Scale bar, 15 µm.

Supplementary movie 3

Phagocytosis of *S. aureus* by neutrophils. Intravital 2-photon microscopy of *S. aureus*-pGFP infected mouse ears (Catchup^{IVM}) starting 4 h p.I.. Scale bar, 10 µm.

Supplementary figure S1. An *in vivo* proliferation biosensor for *S. aureus*.

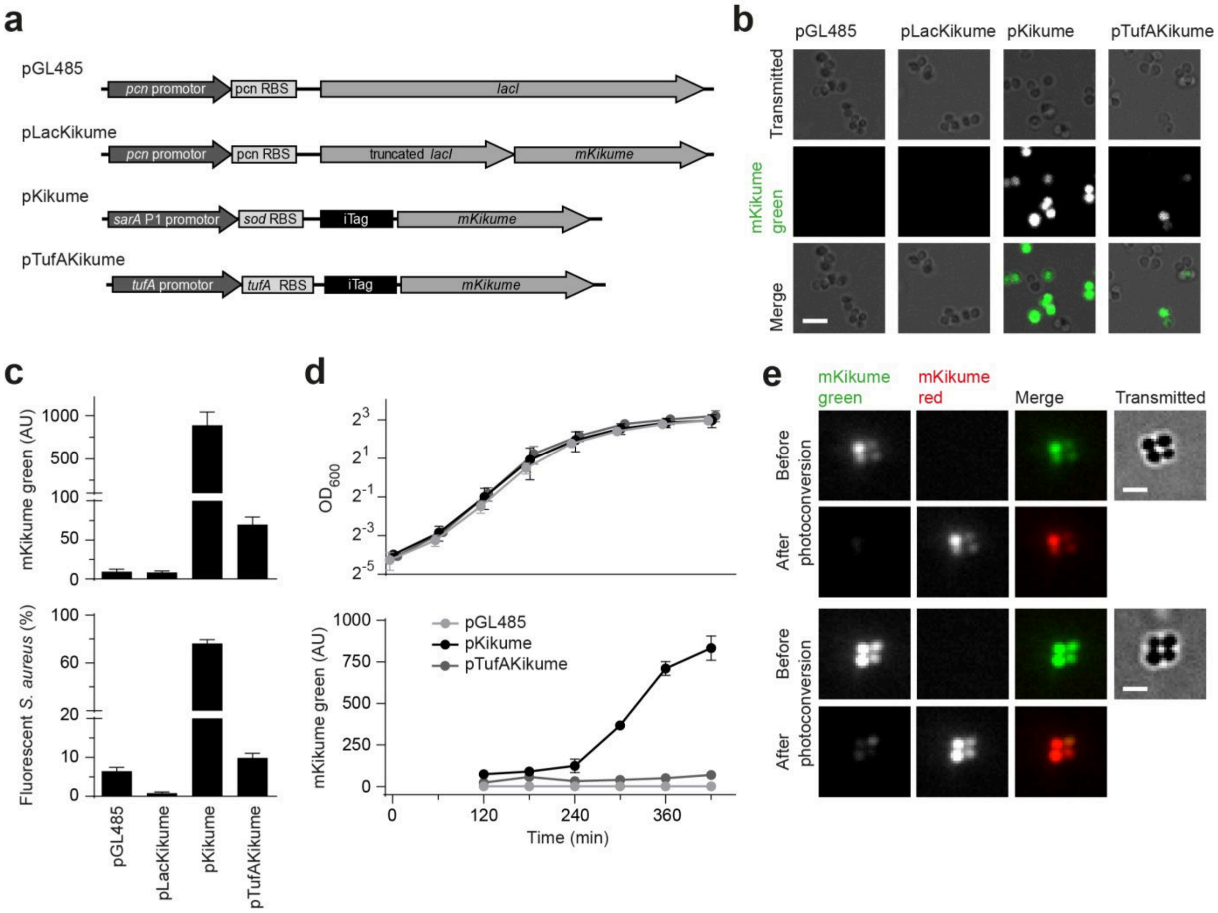

a) Schematic representation of pGL485 derivatives, which were used for mKikume expression in *S. aureus*. *pcn*=penicillinase, RBS=ribosome binding site, *sod*=superoxide dismutase. **b)** Confocal imaging of *S. aureus* cultivated for 7 h with different plasmids in glass bottom dishes. Scale bar, 3 µm. **c)** Flow cytometry analysis. Mean (+/- standard deviation) green fluorescence, representing mKikume signal of four individual 7 h cultures (upper graph). Mean (+/- standard deviation) percentage of green fluorescent bacteria of four individual 7 h cultures (lower graph). **d)** Growth curves of *S. aureus* cultures containing different plasmids. OD_{600} measurement over time of three individual cultures (upper graph). Measurement of green fluorescence, by flow cytometry, over time of three individual cultures (lower graph). **e)** Wide-field microscopy of *S. aureus*-pKikume over night cultures growing on an agar pad before and after photoconversion with violet (405 nm) light. Scale bar, 3 µm.

Supplementary figure S2. Sensitivity of mKikume fluorescence for detecting *S. aureus* in the tissue.

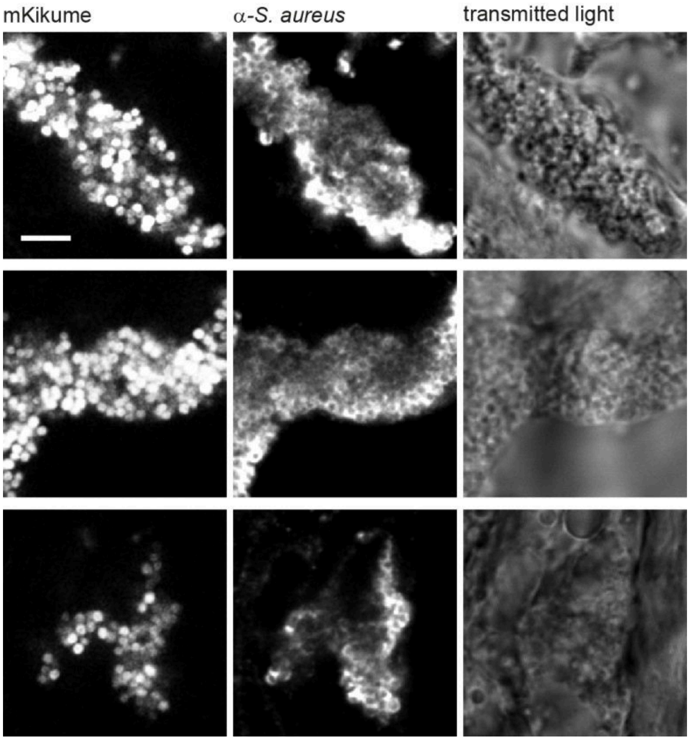

Representative confocal microscopy examples of a *S. aureus*-pKikume infected mouse ear, sectioned and stained against *S. aureus*. Scale bar, 5 µm.

Supplementary figure S3. Tracking *S. aureus* proliferation in the ongoing infection by intravital 2-photon microscopy.

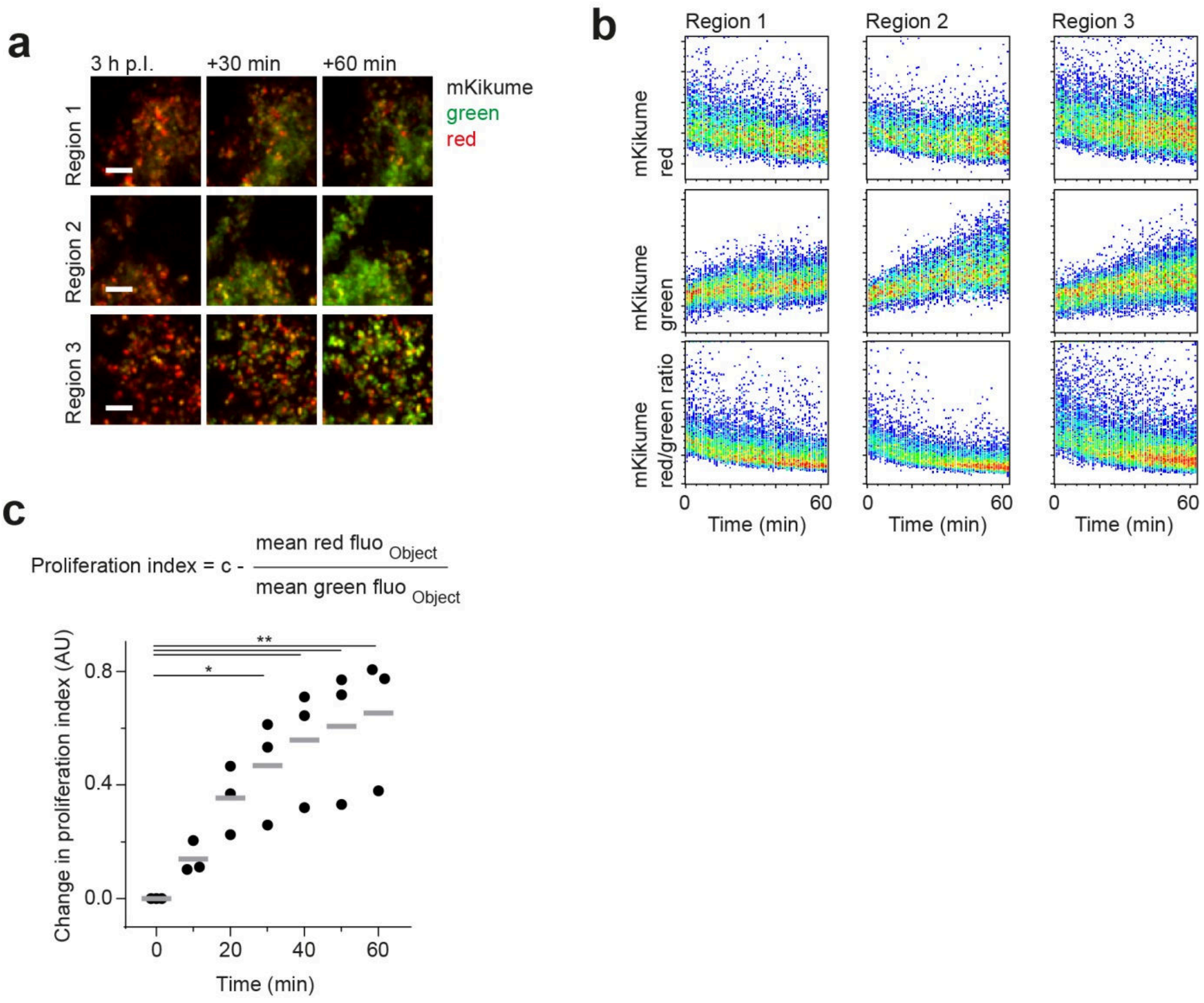

a) Examples of three regions of one intravital 2-photon microscopy of an infected mouse ear starting right after photoconversion 3 h p.I.. Projections of three-dimensional images of 20 Z-slices spaced 2 µm are shown. Scale bar, 10 µm. **b)** Bacteria in the imaged regions shown in a) were automatically 3D-segmented and mean mKikume red and green fluorescence values were extracted for each detected shape and plotted over time. **c)** Red and green fluorescence values were used to calculate a proliferation index for each detected shape. The 80th percentile was calculated and changes compared to the initial value were plotted in 10 min intervals. Each dot indicates a 10 min interval of one region analysed in a) and b). Horizontal bar represent the mean. **, $p<0.01$; *, $p<0.05$ as determined by one-way ANOVA (comparison to the initial value).

Supplementary figure S4. Recruitment of immune cells in *cybb*$^{-/-}$ mice.

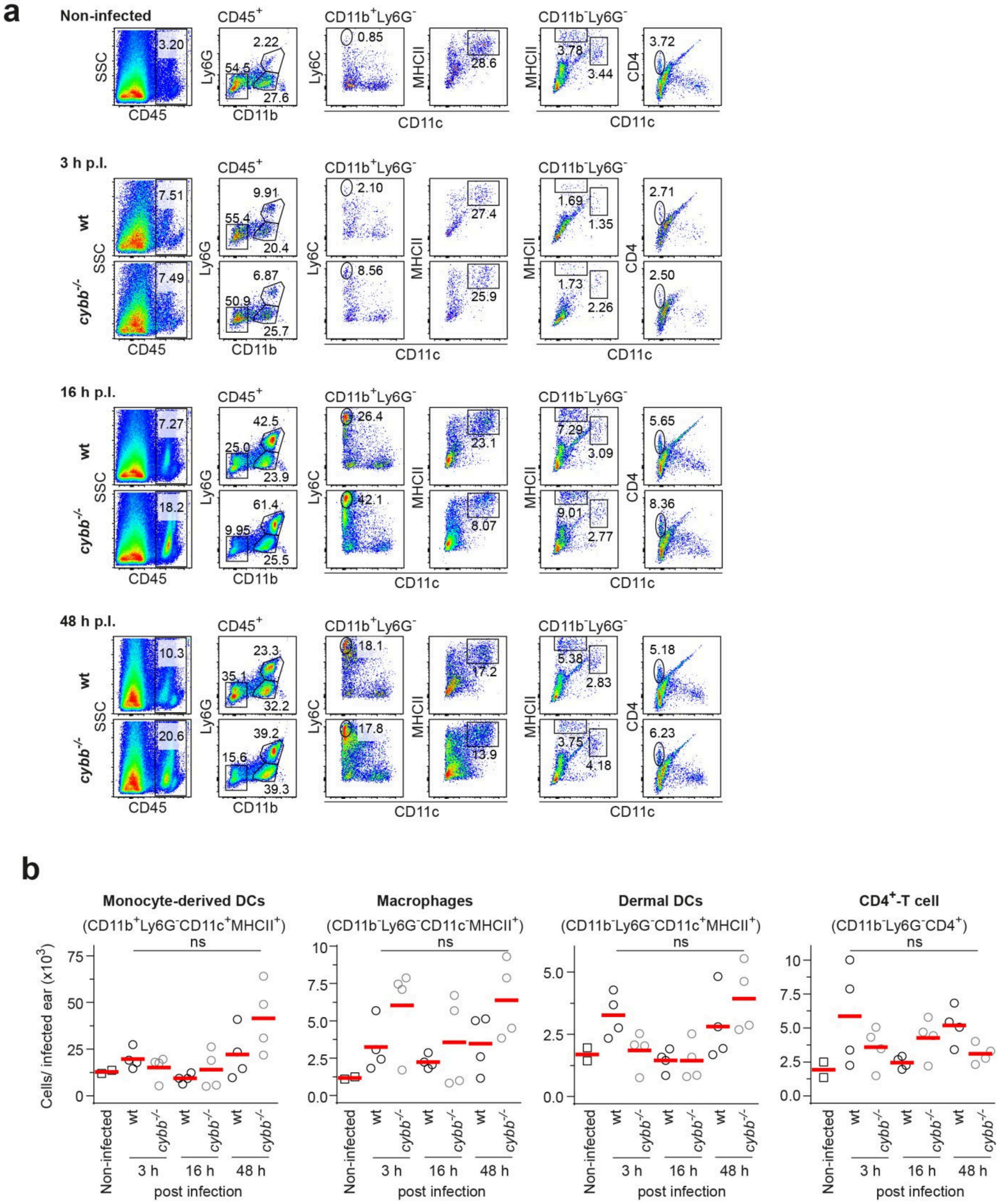

a) Flow cytometry analysis of leukocytes recruited to the site of *S. aureus*-pKikume infection at 3, 16, and 48 h p.I. in wt and *cybb*$^{-/-}$ mice. Data from of non-infected mice are shown for comparison. Data are representative of at least 8 infected ears per condition. **b)** Cells counts in infected ears analysed by flow cytometry and calculated based on counting beads. Gating on CD45^{+} cells, CD4^{+} T-cells (CD11b^{-}, Ly6G^{-}, CD4^{+}), Macrophages (CD11b^{-}, Ly6G^{-}, CD11c^{-}, MHC-II^{+}), Dermal DCs (CD11b^{-}, Ly6G^{-},

CD11c^{+}, MHC-II^{+}) and Monocyte-derived DCs (CD11b^{+}, Ly6G^{-}, CD11c^{+}, MHC-II^{+}). Each dot represents one individual ear; horizontal bars represent the mean; ns, not significant as determined by one-way ANOVA.

Supplementary figure S5. NADPH oxidase-mediated dampening of *S. aureus* recovery from photoconversion in *in vitro*-infected neutrophils.

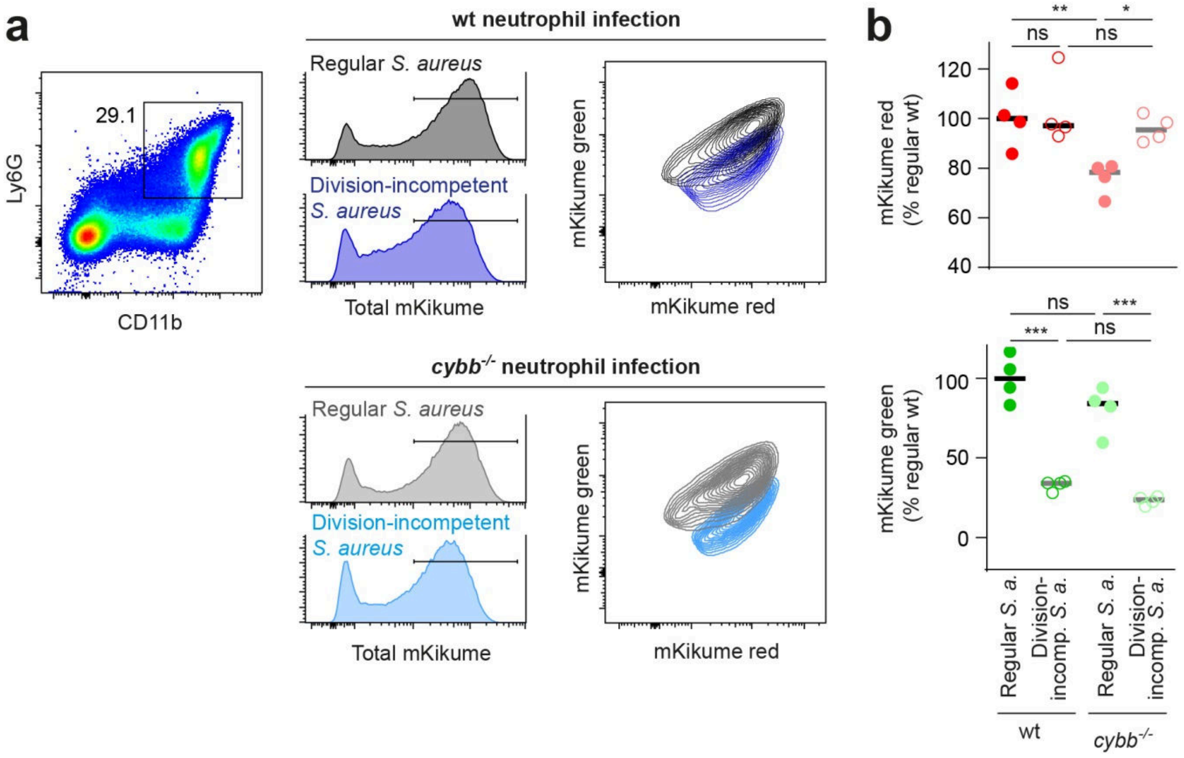

a) Flow cytometry analysis of bone marrow-isolated neutrophils infected regular or division incompetent *S. aureus*-pKikume. Selection of infected neutrophils according to the calculated total mKikume (both green and red) signal and analysis of red and green *S. aureus* mKikume fluorescence 60 min after photoconversion within infected neutrophils. Data are representative of four separate samples acquired from two independent experiments. **b)** Quantitative analysis of mKikume red and green fluorescence 60 min after photoconversion. Each symbol represents one separate sample; horizontal bars represent the median; ***, p<0.001; **, p<0.01; *, p<0.05; ns, not significant according to ANOVA with Bonferroni post-test.

References

1. Habuchi, S., Tsutsui, H., Kochaniak, A.B., Miyawaki, A. & van Oijen, A.M. mKikGR, a monomeric photoswitchable fluorescent protein. *PLoS One* **3**, e3944 (2008).
2. Liew, A.T., *et al.* A simple plasmid-based system that allows rapid generation of tightly controlled gene expression in Staphylococcus aureus. *Microbiology* **157**, 666-676 (2011).
3. Malone, C.L., *et al.* Fluorescent reporters for Staphylococcus aureus. *J Microbiol Methods* **77**, 251-260 (2009).
4. Catalao, M.J., Figueiredo, J., Henriques, M.X., Gomes, J.P. & Filipe, S.R. Optimization of fluorescent tools for cell biology studies in Gram-positive bacteria. *PLoS One* **9**, e113796 (2014).
5. Kreiswirth, B.N., *et al.* The toxic shock syndrome exotoxin structural gene is not detectably transmitted by a prophage. *Nature* **305**, 709-712 (1983).
6. Horsburgh, M.J., *et al.* sigmaB modulates virulence determinant expression and stress resistance: characterization of a functional rsbU strain derived from Staphylococcus aureus 8325-4. *Journal of bacteriology* **184**, 5457-5467 (2002).
7. Kwan, T., Liu, J., DuBow, M., Gros, P. & Pelletier, J. The complete genomes and proteomes of 27 Staphylococcus aureus bacteriophages. *Proc Natl Acad Sci U S A* **102**, 5174-5179 (2005).
8. Hasenberg, M., *et al.* Rapid immunomagnetic negative enrichment of neutrophil granulocytes from murine bone marrow for functional studies in vitro and in vivo. *PLoS One* **6**, e17314 (2011).
